# Imaging of the host/parasite interplay in cutaneous leishmaniasis

**DOI:** 10.1016/j.exppara.2010.05.014

**Published:** 2010-11

**Authors:** Owain R. Millington, Elmarie Myburgh, Jeremy C. Mottram, James Alexander

**Affiliations:** a Centre for Biophotonics, UK; b Strathclyde Institute of Pharmacy & Biomedical Sciences, University of Strathclyde, Glasgow G4 0NR, UK; c Wellcome Trust Centre for Molecular Parasitology and Division of Infection and Immunity, Faculty of Biomedical and Life Sciences, University of Glasgow, 120 University Place, Glasgow G12 8TA, UK

**Keywords:** *Leishmania*, Immunology, Microscopy

## Abstract

An understanding of host–parasite interplay is essential for the development of therapeutics and vaccines. Immunoparasitologists have learned a great deal from ‘conventional’ *in vitro* and *in vivo* approaches, but recent developments in imaging technologies have provided us (immunologists and parasitologists) with the ability to ask new and exciting questions about the dynamic nature of the parasite–immune system interface. These studies are providing us with new insights into the mechanisms involved in the initiation of a *Leishmania* infection and the consequent induction and regulation of the immune response. Here, we review some of the recent developments and discuss how these observations can be further developed to understand the immunology of cutaneous *Leishmania* infection *in vivo*.

## 1 Introduction

*Leishmania* infections present a major global health challenge, with an estimated 12 million people infected worldwide and consequently there is an urgent need for better prophylactic and therapeutic interventions for this disease (Mansueto et al., 2007). The induction of an immune response requires a series of complex interactions between several different cell types (and their products), with an orchestra of secreted proteins, surface receptors, signalling pathways and intracellular processes occurring in a highly specialised manner (Guermonprez et al., 2002). Importantly, relatively small changes in only a few of these cell–cell or protein–protein interactions can dramatically alter the functional characteristics of the immune response generated (Gett et al., 2003), and can dictate whether an infection is controlled or establishes a chronic, progressive disease in the host. *Leishmania* is of particular interest in this regard since it is a major global health challenge in its own right, but relatively few differences between individual species (e.g. *Leishmania major* versus *Leishmania donovani* versus *Leishmania braziliensis*), or the genetic background and/or immunological status of the host can result in significantly different outcomes (cutaneous versus visceral, chronic versus healing) (Ivens et al., 2005; McMahon-Pratt and Alexander, 2004; Peacock et al., 2007; Sacks and Noben-Trauth, 2002). Hence, in order to develop novel therapeutics and vaccine strategies, an improved understanding of the underlying immunology is needed. The wide spectrum of human disease can be mimicked in murine models and these have been used extensively for than conventional imaging *in vitro* studies to dissect host cell/parasite interactions, as well as *in vivo* to elucidate the complex nature of the immunological process involved. Critically, the complex nature of the immune system necessitates the use of *in vivo* models of infection in order to elucidate the precise roles of the specific interactions involved.

Typically, the study of *Leishmania* infections in the mouse has used approaches such as parasite-specific immunoglobulins, measurement of lesion size, parasite burden and *in vitro* recall responses of lymph node or spleen cells to understand the fundamental immunology of infection. As novel techniques developed allowing in-depth analysis of the specific cells of the immune system, immunoparasitologists have embraced these approaches to better understand the kinetics of *Leishmania* infection.

Thus, flow cytometric analysis has been used to investigate many aspects of *Leishmania* infection, such as the role of CC chemokine receptor-2 in the migration of skin-derived dendritic cells (DCs) to the lymph node, antigen presentation by tissue-derived versus lymph node-resident DCs, as well as to examine the kinetics of cell recruitment back into the lesion (Belkaid et al., 2000; Iezzi et al., 2006; Sato et al., 2000). Others used immunohistochemical and immunofluorescence staining of sections taken at different times following *Leishmania* infection to establish the distinct pathways involved in mediating immunity, migration and disease regulation (Ato et al., 2006). However, recent advances in imaging systems have provided the opportunity to further understand the development of infection and the induction of an immune response against and its modulation by the parasite.

## 2 Background to imaging approaches

Ever since van Leeuwenhoek developed his simple microscope and observed protozoa in droplets of water, researchers have been fascinated with the possibility of visualising the interactions of parasites and the immune system. The use of fluorescence- or luminescence-based imaging approaches has allowed immunoparasitologists to characterise many of the fundamental processes involved in *Leishmania* infection. Whilst the use of green fluorescent protein (GFP) and other fluorochromes has expedited the use of fluorescence microscopy, the development of imaging systems allowing users to look across a variety of scales (from full animal imaging down to the sub-micron resolution) has been of critical importance. There have been several recent developments providing novel approaches to assess cell–cell or protein–protein interactions, visualise single molecules and offer imaging beyond the diffraction limit.

### 2.1 Bioluminescence

*In vivo* bioluminescence imaging was first used to monitor infection in a *Salmonella typhimurium* model (Contag et al., 1995). By introducing firefly or *Renilla* luciferase into pathogens or cells of interest their *in vivo* localisation and proliferation or clearance can be assessed. Interaction between the luciferase enzyme and its substrate (luciferin) results in emission of photons, which can then be detected using a high sensitivity cooled charge-coupled device (CCCD) camera. This approach allows whole-body imaging of small animals and researchers are able to localise the signal by overlaying a map of specific tissues and organs. Indeed, some bioluminescence imaging systems now include an integrated X-ray system to provide further locational detail (Andreev et al., 2007; Backer et al., 2007). However, significant absorption of light by body tissues means that these approaches rely on a relatively strong signal, requiring a minimum of 10^3^–10^4^ reporter cells to generate sufficient signal for detection (around 20 pg of luciferase (Lang et al., 2005; Rettig et al., 2006)). The recent development of red-shifted luciferases will aid in this enhancing this sensitivity, reducing the light absorption by tissue (Loening et al., 2007; Shapiro et al., 2005). Also, whilst these systems provide some magnification capability, (typically resolving down to sub-millimetre resolution), their resolution and sensitivity does not provide the opportunity for analysing the behaviour of single cells or small populations of cells. A major disadvantage of bioluminescent proteins is their requirement for available substrate. This is especially problematic for long term *in vivo* experiments where continuous injection of substrate would be needed. Furthermore, non-homogenous distribution of substrate *in vivo* differs depending on the route of substrate administration and complicates the accurate localisation of bioluminescent cells or pathogens (Claes et al., 2009).

### 2.2 Fluorescence microscopy

Unlike bioluminescence (where an enzymatic reaction generates emitted photons), fluorescence microscopy introduces illumination of one wavelength to excite a fluorescent molecule and then detects photons emitted at a red-shifted wavelength as the excited electrons decay back to their ground state. Several advances in fluorescent microscopy have provided researchers with a wide range of facilities for imaging, depending on the needs of the user.

Epi-fluorescence microscopy provides an excellent introduction to imaging, using relatively broad excitation and detection systems to capture emissions from much of the depth of the sample. Whilst this provides the opportunity to visualise cells and parasites with good lateral resolution (e.g. Kupfer et al. (1986), Monks et al. (1998)), there is little control over the focal plane examined and deconvolution is often required for effective 3-dimensional analysis. Using confocal microscopy allows researchers to image in 3 and 4 dimensions with much more precision. By using a pinhole to restrict light collection to a single focal plane provides much greater axial resolution and provides the opportunity to resolve images down to the sub-micron level. For example, confocal microscopy has been used to provide high-resolution images of *Leishmania-*containing phagosomes to assess the localisation of MHC molecules (Flohe et al., 1997; Lang et al., 1994), actin polymerisation (Holm et al., 2001) and disruption of lipid rafts (Chakraborty et al., 2005). However, relatively high laser intensities are required for these imaging approaches, which can result in damage to biological samples and photobleaching above and below the focal point. The spinning disk confocal microscope permits higher speed imaging and is associated with less tissue damage, providing the opportunity for *in vivo* imaging of fluorescent samples. Whilst these systems have been used to image cells of the immune system with some success (e.g. Pflicke and Sixt (2009)), an inherent problem with confocal imaging systems is the limited depth penetration associated with light scattering by the tissue (especially at lower wavelengths).

An important development was that of multiphoton laser-scanning microscopy (MPLSM; (Squirrell et al., 1999)). This provides the 3 and 4-dimensional analysis of confocal imaging approaches, but rather than exciting fluorescence throughout the depth of the tissue, the laser excitation is focused to a very tight point. Importantly, fluorophores are stimulated by the near-simultaneous excitation of two higher wavelength photons (as opposed to a single lower wavelength photon in confocal imaging) (Squirrell et al., 1999). This allows imaging of fluorescent samples at depths in the order of hundreds of microns within the tissue, with relatively little damage to surrounding tissues. However, this approach often requires removal (or surgical exposure) of the tissue of interest, with limited possibility for repeated imaging. Also, the relatively small field-of-view restricts the area of imaging to a few hundred microns across and whilst MPLSM is able to probe deeper into tissue than previous approaches, organs of interest can be much thicker than light penetration allows.

**Fig. 1:**
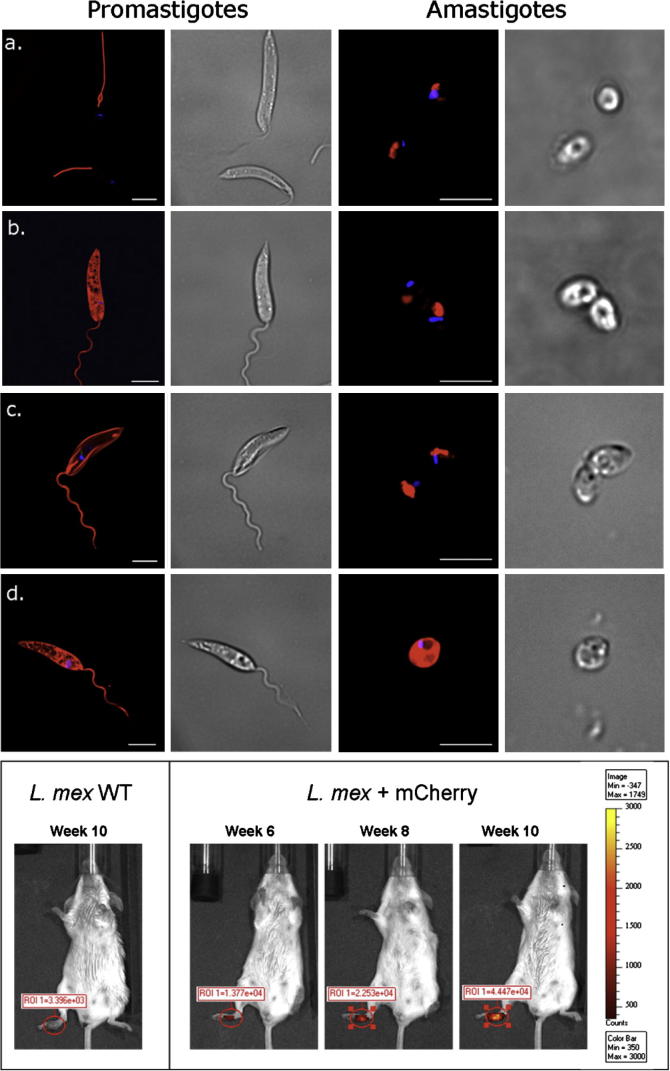
Imaging of *Leishmania mexicana* expressing red fluorescent proteins. Eα-DSRed (a + b) or Eα-mCherry (c + d) were fused to the HASPB or HASPB G/A mutant signal sequence to direct fusion proteins to the surface or cytosol of *L. mexicana* parasites (Denny et al., 2000; Prickett et al., 2006). Live promastigotes (left) or lesion-derived amastigotes (right) were imaged on a Deltavision epifluorescent microscope. (a) Tetrameric DSRed fused to HASPB signal is not detected on the surface of promastigotes or lesion-derived amastigotes and appears to mislocalise to the lysosome. (b) DSRed fused to HASPB G/A is visible in the cytosol of promastigotes but appears to localise to large lysosomes/megasomes in amastigotes. (c) The monomeric protein, mCherry, fused to HASPB is expressed on the promastigote surface but appears to locate to lysosomes/megasomes in amastigotes. (d) mCherry fused to HASPB G/A is expressed in the cytosol of both promastigotes and lesion-derived amastigotes. Kinetoplast DNA (blue) is stained with DAPI. Corresponding brightfield images are shown on the right of each epifluorescent image. Scale bar = 5 μm. (e) Stationary-phase *L. mexicana* promastigotes expressing Eα-mCherry fused to the HASPB G/A mutant signal were injected into right hind footpads of BALB/c mice. Disease progression was monitored by *in vivo* fluorescence imaging using a Xenogen IVIS Spectrum. The Region of Interest (ROI) indicates fluorescence measured in the footpad at each time point after infection. Fluorescence increased over time and corresponded to an increase in lesion size and parasite load. Mice infected with wild-type *L. mexicana* (WT) showed background levels of fluorescence while lesion size progressed over time. (For interpretation of the references in colour in this figure legend, the reader is referred to the web version of this article.)

Whilst these approaches have proved incredibly useful in terms of understanding the host–parasite interaction of *Leishmania* infection, it is important to understand some of the problems associated with these imaging approaches. For example, bioluminescence imaging of luciferase-expressing parasites requires efficient uptake and delivery of the luciferin substrate to parasites contained within the parasitophorous vacuole. In addition, the acidic pH of the parasitophorous vacuole may alter the emission spectra of certain fluorochromes (Tsien, 1998). The *Leishmania* life cycle is complex, and certain stages may be associated with rearrangement of proteins, resulting reporter fluorochromes localising to different compartments in promastigotes and amastigotes. For example, we have generated *Leishmania mexicana* parasites expressing Eα-DSRed and Eα-mCherry with either a HASPB or HASPB G/A mutant signal sequence to localise the fluorochromes to the parasite membrane or to retain the protein in the cytosol (Fig. 1). In these parasites, stage-specific differences can be observed in terms of the localisation of the fluorochromes, with DsRed correctly targeting to the cytosol of promastigotes, but localising in lysosomes/megasomes during amastigote stages (Fig. 1b). Interestingly, differences between in the membrane targeting of tetrameric DSRed and monomeric mCherry are also evident (Fig. 1a–d), demonstrating the importance of appropriate fluorochrome selection. In addition, over-expression of fluorescent/bioluminescence reporter proteins may influence parasite fitness, with EGFP-expressing *L. mexicana* showing delayed lesion development compared with wild-type parasites (Bennett et al., 2001).

## 3 What imaging has told us about *Leishmania* infection and the immune response?

### 3.1 Visualising the complexity of intracellular infection

**Fig. 2:**
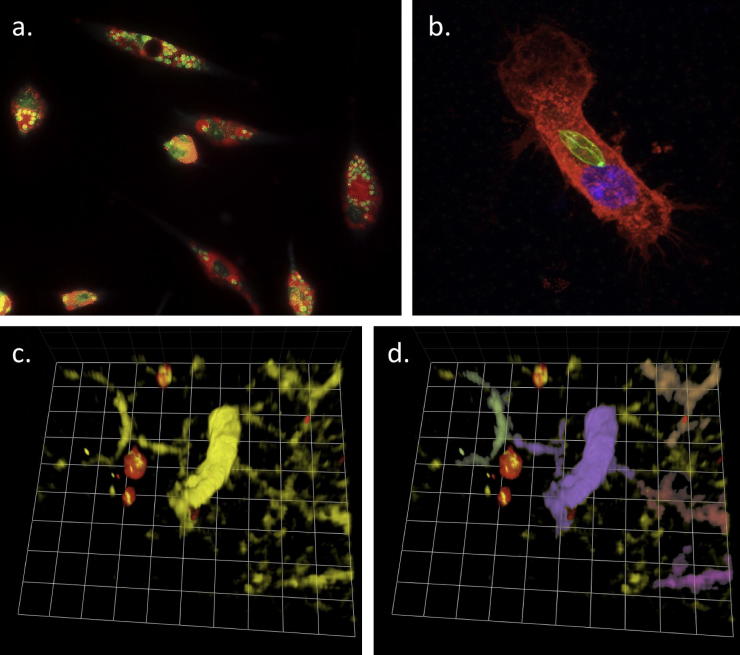
The evolution of fluorescence microscopy in studies of *Leishmania*. (a) Dark-field vital fluorescence staining of macrophage secondary lysosomes using acridine orange. Lysosomes have fused to parasitophorous vacuoles, some containing single parasites and others multiple parasites. (b) Three-dimensional confocal imaging of GFP-expressing *L. mexicana* (green) internalised by a dendritic cell (stained with cholera toxin subunit B-AF647 to reveal lipid rafts; red). GFP-expressing promastigotes and dendritic cells were co-cultured for 1 h to allow internalization of the parasite prior to fixation and imaging. (c and d) *In vivo* imaging of dendritic cell/*Leishmania* interaction by multiphoton imaging the ear of a CD11c-YFP mouse infected with mCherry-expressing *L. mexicana*. The image shows a number of dendritic cells (yellow) interacting with fluorescent parasites (red) *in vivo*. Using Volocity analysis software (Improvision), individual cells can be identified and false-coloured (d), allowing tracking over time. Grid square = 8 μm. (For interpretation of the references in colour in this figure legend, the reader is referred to the web version of this article.)

Early electron microscopy and fluorescence studies of intracellular *Leishmania* infection provided an insight into the complex mechanisms of uptake and movement of parasite-containing vacuoles within an infected macrophage. Thus, it was demonstrated that amastigotes reside in a phagolysosome but that a parasite-induced manipulation of the process of phagosome fusion could enhance parasite growth (see Fig. 2a) (Alexander, 1981). These early observations suggested a defect in phagosome–endosome fusion following promastigote infection. Phagosomal development is typically associated with accumulation of *F*-actin, which is then removed as the phagosome matures (Aderem and Underhill, 1999). Using confocal microscopy to visualise GFP-expressing wild-type and LPG-deficient promastigotes, Holm et al. demonstrated that LPG plays an important role in maintaining this periphagosomal F-actin, which has been proposed to provide a physical barrier to reduce phagosome–endosome fusion (Holm et al., 2001). More recently, studies have used fluorescence systems to assess the maturation of parasite-containing phagosomes. Rab5 is a key regulator of phagosome maturation, and using cells derived from GFP-Rab5 transgenic mice Lippuner et al. were able to assess whether *L. mexicana* are contained within Rab5^+^ compartments in macrophages (Lippuner et al., 2009). Real-time fluorescence imaging of these cultures conclusively demonstrated that *L. mexicana*-containing phagosomes were Rab5^+^ for much shorter times than other phagosomes and that this was associated with LPG expression on the surface of the parasite. This demonstrates the importance of analysing these complex spatial and temporal cell–pathogen interactions over a significant period of time. Real et al. (2008) made use of GFP-expressing *Leishmania amazonensis* parasites to show the fusion of newly-formed parasite-containing phagosomes (or PV) to pre-established PV’s containing unlabelled parasites (Real et al., 2008). Their model may be useful for future studies on the parasite or host factors that influence PV fusogenicity and maturation.

The use of fluorescent parasites is also helping our understanding of the fundamental biology of *Leishmania* infection (Fig. 2b). Using transgenic *L. amazonensis* parasites expressing the DsRed2 fluorescent protein, Lecoeur et al. (2009) investigated modulation of dendritic cell gene expression by *Leishmania*. Several reports have used fluorescent parasites to investigate the effects of *Leishmania* upon dendritic cells (e.g. Bennett et al. (2001), Colmenares et al. (2002), Gorak et al. (1998), Iezzi et al. (2006), Jebbari et al. (2002), Maroof and Kaye (2008), Misslitz et al. (2004), Prina et al. (2004), von Stebut et al. (1998), Williams (1988)), but purification of parasite-containing dendritic cells (based on fluorescence) revealed significant modulation of transcription of several genes critical for DC function, which was not identified in the unsorted population. Importantly, whilst epi-fluorescence imaging was also used to confirm that the DsRed-positive cells contained fluorescent parasites, in the future it will be important to develop *in situ* visualisation and purification procedures to ensure that cells do not acquire parasites during tissue processing. The use of these approaches to specifically identify parasite-containing cells will be of great importance in the development of high-throughput screening approaches currently under development.

### 3.2 Analysing *Leishmania* infection and cell recruitment *in vivo*

The complex and dynamic nature of the *Leishmania* infection site necessitates *in vivo* studies to characterise the kinetics of the parasite burden and the associated recruitment and function of key cells of the immune system. Several fluorescence-based approaches have been employed to visualise, at a cellular level, the response to infection and the mechanisms involved (Fig. 2c and d). Recently, Reinhardt et al. (2009) have used fluorescent reporter mice to analyse the cytokine response to *L. major* infection. Using flow cytometry and immunofluorescence in strains of mice which reveal active production of IL-4 and of IFNγ they showed that, following *L. major* infection, IL-4 release is essentially limited to B cell follicles and germinal centres. Importantly, by isolating cell conjugates based on fluorescence-reporters of cytokine production, the authors demonstrated that both the IL-4-producing and IFNγ-producing follicular helper T cells interacted with B cells and specifically direct immunoglobulin switching towards IgG1 and IgG2a, respectively.

Obviously, the changing environment of the *Leishmania* infection site necessitates an imaging modality that allows repeated imaging sessions in order to monitor these changes. The development of bioluminescent *L. amazonensis* provided a sensitive detection method to monitor growth over several weeks following infection. Using luciferase-transfected parasites, Lang et al. monitored parasite development at the site of intradermal inoculation and in the draining lymph node, demonstrating that this approach provides a highly sensitive method for detection and quantification of early infection (Lang et al., 2005). Importantly, this approach negated the need to sacrifice animals at regular time points following infection, allowing repeated quantification of parasite numbers in the same animal over time. Recently, we have used mCherry-expressing *L. mexicana* parasites to monitor development of cutaneous infection over time (Fig. 1e). Combinations of these approaches will allow researchers to visualise the recruitment of specific cell types during infection, as has recently been investigated in visceral leishmaniasis (Beattie et al., 2010).

### 3.3 Visualising the dynamic nature of cells of the immune system during *Leishmania* infection

With the advent of MPLSM, immunologists are now able to appreciate the complex and dynamic nature of an immune response in unprecedented detail. Whilst previous reports had characterised recruitment of inflammatory cells into the site of infection using flow cytometry and immunofluorescence, we are now able to examine the spatial and temporal nature of the response to *Leishmania* infection.

Several reports have described the role of neutrophils in the early response to experimental *Leishmania* infection following needle inoculation. Indeed, work by Tacchini-Cottier et al. demonstrated the significance of these cells, which are recruited within an hour post-infection and, through early production of IL-12, are associated with the resistance/susceptibility of different strains of mice (Tacchini-Cottier et al., 2000). However, it is clear that sand fly biting is also associated with tissue damage and initiates a strong inflammatory response, even in the absence of parasites (Andrade et al., 2007). By using MPLSM to image the early response to infection, Peters et al. visualised the migration of neutrophils in mice expressing GFP under the lysozyme M promoter (Peters et al., 2008). They demonstrated that following the sand fly bite, neutrophils are rapidly recruited to the site of injury (irrespective of the presence of parasites) and form a “plug” in order to close over the hole left by the sand fly proboscis. Subsequently, the fast-moving neutrophils migrate to the site of dermal infection, take up *Leishmania* parasites by phagocytosis and reduce their migratory capacity. Critically, despite phagocytosis, parasites remained viable and transfer of infected neutrophils into naïve animals was able to transfer infection which was associated with the transfer of parasites from neutrophils to macrophages. Hence, the initial uptake of parasites by neutrophils may be important in the recruitment and subsequent modulation of macrophages to provide the parasite with an ideal host cell.

Concomitantly with recruitment of neutrophils from blood vessels, dermal DCs begin to respond to *Leishmania* infection. Whilst the early reports proposed a role for Langerhans cells in the response to *Leishmania* infection (Moll et al., 1993), several recent reports have suggested that (at least in *L. major* infection) these cells are not necessary for the activation of CD4 T cell responses in the draining lymph node (Lemos et al., 2004; Ritter et al., 2004). Instead, it has been suggested that activation of local dermal DCs may be important in the induction of the adaptive immune response (Ritter et al., 2004). Hence, a recent report by Ng et al. investigated the *in vivo* response of Langerhans cells (LC) and dermal DCs (DDCs) following infection with *L. major* (Ng et al., 2008). Using CD11c-YFP mice, the authors demonstrated that LC and DDCs showed distinct behaviours in steady-state, with epidermal LCs remaining relatively static but extending long dendrites to effectively survey the microenvironment. Conversely, DDCs actively migrated through the interstitial space of naïve animals, allowing them to rapidly detect and respond to infection. Thus, within 20 min of *L. major* inoculation, DDCs decreased their migration and phagocytosed live parasites. Interestingly, this change in behaviour was not restricted to DCs interacting with parasites, suggesting that the localised inflammation alters DDC migratory capacity rather than parasite uptake alone. How the migratory arrest of these cells in the tissue correlates with migration to the draining lymph node is unclear (Ritter et al., 2004), but may explain the relative importance of lymph node-resident DCs in antigen-presentation *in vivo* (Iezzi et al., 2006).

As highlighted above, the application of MPLSM has provided an insight into the interactions involved in the activation of naïve T cell responses by antigen-presenting cells in the lymph node and enhanced our appreciation of the dynamic nature of naïve T cells (Miller et al., 2002). Recent reports have investigated the behaviour of T cells during a recall response in the lymph node following challenge with either model antigen or *Toxoplasma gondii* (Chtanova et al., 2009; Schaeffer et al., 2009; Wilson et al., 2009). However, the behaviour of activated T cells in peripheral tissues has, until recently, remained ill-defined. The generation of transgenic mice expressing a T cell receptor specifically recognising the LACK antigen has allowed immunoparasitologists to examine many of the features associated with the activation of *Leishmania*-specific adaptive immunity (Reiner et al., 1998; Wang et al., 2001). Thus, Filipe-Santos et al. have recently used these LACK-specific T cells to examine interactions between activated T cells and parasite-containing cells in the *Leishmania* lesion (Filipe-Santos et al., 2009). By imaging activated CFSE-labelled LACK-specific WT15 CD4^+^ T cells as well as polyclonal (labelled with both SNARF-1 and CFSE), it was demonstrated that whilst activated T cells were recruited into the site of infection non-specifically, only the parasite-specific T cells altered their behaviour. The LACK-specific T cells were observed to slow down and interact with some parasite-containing cells (identified by DsRed-expressing *L. major*). Interestingly, not all infected cells were able to attract T cells and areas containing a high density of parasites were impenetrable to T cells. Whether this represents differences in presentation of antigens, production of inflammatory cytokines or chemokines, or simply physical constraints in the microenvironment due to other (unseen) cells, remains to be established.

NK cells have an important role in host protection. In addition to their cytotoxicity role, recent evidence suggests an important function of NK cells is in influencing DC function and T cell effector responses (Ferlazzo et al., 2002; Gerosa et al., 2002; Piccioli et al., 2002). By visualising the interactions between NK cells, DCs, and T cells, Bajenoff et al. characterised an important role of early NK-derived IFNγ in directing the polarisation of CD4 T cell response (Bajenoff et al., 2006). Using MPLSM, the authors demonstrated that lymph node-resident NK cells are relatively slow-moving and establish long interactions with DCs even in the resting state, but that following *L. major* infection, NK cells are recruited to the paracortex of the lymph node and associate with parasite-specific CD4 T cells and DCs. Thus, by imaging NK cell behaviour in a *Leishmania* model, a potential role for these multi-cellular interactions in influencing the immune response was identified. The significance of these NK cells in polarising a Th1 response in this system remains to be identified, especially given the lack of requirement for NK cells in Th1-mediated protection from *L. major* infection (Satoskar et al., 1999).

### 3.4 Application of imaging approaches to developing therapeutics and vaccines

Whilst the above reports have provided a fundamental understanding of the biology of *Leishmania* infection, imaging approaches have recently been used to analyse therapeutic and vaccine candidates. A number of groups have used GFP or luciferase-expressing *Leishmania* to assess drug efficacy *in vitro*, often using rapid techniques such as flow cytometry or microplate-based bioluminescence (Dube et al., 2009)*.* These approaches allow a much higher throughput screening for investigating potential drug candidates than conventional imaging, and GFP-expressing *L. major* has been used to screen a large panel of extracts for effects on parasite viability (Plock et al., 2001). Significantly, the ability to introduce GFP or luciferase expression in field-isolates of *L. donovani* allows *in vitro* screening of anti-leishmanial drugs (Ashutosh et al., 2005; Singh and Dube, 2004).

Fluorescent and luminescent parasites are also being applied for *in vivo* analysis of therapeutic and vaccine development. Using a GFP-expressing *L. amazonensis*, Demicheli et al. showed that β-cyclodextrin enhances the uptake of orally-delivered antimonials, with significantly reduced parasite loads (analysed *ex vivo*). Lecoeur et al. demonstrated the feasibility of using luciferase-expressing bioluminescent *L. major* to assess topical therapeutics directly *in vivo* (Lecoeur et al., 2007). Application of WR279396 (a recently developed aminoglycoside formulation shown to be effective in localised cutaneous leishmaniasis) to infection sites reduced the bioluminescence signal and using whole-body imaging analysis provided a more sensitive approach than conventional clinical monitoring. In a similar approach, Mehta et al. used episomal expression of GFP in *L. amazonensis* and analysed whole-body fluorescence to demonstrate the efficacy of a vaccine candidate (Mehta et al., 2008). Their work suggested that this approach provided an increased sensitivity compared with standard approaches, as well as providing real-time data on the distribution of infection.

Whilst we have focused here on imaging the interactions between the immune system and *Leishmania* parasites, several groups have developed approaches for understanding the development of parasites in the sandfly host. Thus, Kimblin et al. (2008) infected sandflies with RFP-expressing *L. major* parasites and tracked infection in flies by imaging whole sandflies (Kimblin et al., 2008). In Since Akopyants et al. (2009) have demonstrated genetic exchange between *Leishmania major* parasites in the sand fly vector (Akopyants et al., 2009), fluorescent and bioluminescent markers could also be used to track organisms of different lineages and their genetic recombination, as has been shown with *Trypanosoma* (Peacock et al., 2009). These studies open new possibilities for developing approaches to target parasites in the vector, rather than in the mammalian host.

## 4 The future’s bright

Clearly the above approaches have provided a much greater understanding of the fundamental biology of *Leishmania* infection in the mouse, as well as starting to provide the potential for developing novel therapeutics and vaccines. The next step is for immunoparasitologists to dissect the many molecular pathways that are important in each of these distinct stages of the immune response and to establish their significance *in vivo*. Obviously, many studies have performed large-scale genomic/proteomic analyses or made use of transgenic and knockout animals to understand the roles of specific cytokines and receptors, as well as developing cell-specific expression/depletion mice to begin to understand the complex (and often pleiotropic) roles of specific molecules in infection. To fully comprehend the pathways involved we will need to combine these approaches with imaging systems to assess the distinct behaviour of specific cell types in these altered environments, or to use reporters of cell function *in vivo*. However, these approaches pose some significant challenges for biologists, chemists, physicists and engineers. The methods described above have distinct limitations in their ability to resolve less than several hundred nanometres. Thus, application of new and emerging technologies providing resolution below the diffraction limit such as stimulated emission depletion microscopy (STED (Willig et al., 2006), photo-activated localisation microscopy (PALM (Betzig et al., 2006)), stochastic optical reconstruction microscopy (STORM (Rust et al., 2006)) and single molecule imaging (Funatsu et al., 1995) will allow an illuminating insight into the interactions involved in parasite uptake, survival and manipulation of the microenvironment within the parasitophorous vacuole. In parallel, development of novel fluorochromes with emissions further into the red allows improved *in vivo* imaging, with enhanced penetration of excitation and emission light and reduced autofluorescence (Lin et al., 2009; Shu et al., 2009). Critically, it will be important to develop systems that provide the opportunity for a functional read-out *in vivo*. Thus the developments of fluorescent molecules that can be switched on or off and/or change in colour in a controlled way (Chudakov et al., 2004; Marchant et al., 2001) is of particular interest and combination of these developments with transgenic/knockout/knockin animals will contribute to the ‘toolbox’ available for future immunoparasitology studies. The ability to directly manipulate the behaviour of a conscious animal by optically stimulating neurons highlights the incredible potential of these approaches (Airan et al., 2009). Furthermore, the development of alternative *in vivo* imaging approaches such as those based on MRI, positron emission tomography (PET), Raman scattering or microendoscopy provide the exciting possibility of visualising parasite and cellular behaviour without the need for fluorescent labels or without the need for surgical approaches. Critically, the combination of multiple imaging approaches into one (e.g. Olson et al. (2010)), along with other fields, such as genomics, proteomics and metabolomics, will provide the opportunity to identify key pathways that may be useful for development of chemotherapeutics. As highlighted above, imaging approaches are now being adapted for use in the screening of candidate molecules, developing the capacity for the high-throughput screening required if successful therapeutic and vaccination strategies are to be developed further.
